# Microstructure and Mechanical and Corrosion Behavior of Novel High-Entropy CoCrFeNiSiV_x_ (x = 0.25; 0.5; 0.75; 1.0) Alloys

**DOI:** 10.3390/ma18194616

**Published:** 2025-10-06

**Authors:** Rafał Babilas, Monika Spilka, Katarzyna Młynarek-Żak, Adrian Radoń, Wojciech Łoński, Krzysztof Matus, Jakub Bicz

**Affiliations:** 1 Department of Engineering Materials and Biomaterials, Silesian University of Technology, Konarskiego 18a St., 44-100 Gliwice, Poland; monika.spilka@polsl.pl (M.S.); jakubic839@student.polsl.pl (J.B.); 2 Department of Engineering Processes Automation and Integrated Manufacturing Systems, Silesian University of Technology, Konarskiego 18a St., 44-100 Gliwice, Poland; katarzyna.mlynarek-zak@polsl.pl; 3 Łukasiewicz Research Network—Institute of Non-Ferrous Metals, Sowińskiego 5 St., 44-100 Gliwice, Poland; adrian.radon@imn.lukasiewicz.gov.pl; 4 Łukasiewicz Research Network—Institute for Sustainable Technologies, Pułaskiego 6/10 St., 26-600 Radom, Poland; wojciech.lonski@itee.lukasiewicz.gov.pl; 5 Materials Research Laboratory, Faculty of Mechanical Engineering, Silesian University of Technology, Konarskiego 18a St., 44-100 Gliwice, Poland; krzysztof.matus@polsl.pl

**Keywords:** high-entropy intermetallic compounds, corrosion resistance, electrochemical impedance spectroscopy, hardness, tribological properties

## Abstract

In this work, a series of novel high-entropy alloys CoCrFeNiSiV_x_ (x = 0.25; 0.5; 0.75; 1.0) with an intermetallic compound structure was proposed. The effect of vanadium addition on the structure, as well as selected mechanical and corrosion properties, was investigated. In the case of the CoCrFeNiSiV_0.25_ alloy, the structural analysis revealed the formation of a dual-phase structure consisting of Fe_1.812_V_0.907_Si_0.906_-type and Fe_5_Ni_3_Si_2_-type intermetallic phases. The increase in vanadium concentration results in the crystallization of one Fe_1.812_V_0.907_Si_0.906_ intermetallic phase detected by the X-ray diffraction method. The increase in vanadium content had a beneficial influence on the corrosion resistance of CoCrFeNiSiV_x_ alloys in 3.5% NaCl. The CoCrFeNiSiV alloy exhibited the lowest corrosion current density of 0.17 μA/cm^2^ and the highest corrosion potential of −0.228 V. The hardness of the alloys investigated increased with vanadium content, reaching 1006 HV for the equimolar alloy. In turn, the lowest friction coefficient of 0.63 ± 0.06 was obtained for the CoCrFeNiSiV_0.75_ alloy. The abrasive, fatigue, and oxidative wear were identified as the main wear mechanisms.

## 1. Introduction

High-entropy alloys (HEAs) have garnered considerable interest since their first preparation in 2004 [1,2], due to their exceptional versatility resulting from their attractive mechanical and physicochemical properties. These properties make HEAs promising materials for a broad range of applications and different operating conditions, from extreme temperatures to corrosive environments and substantial mechanical loads [3,4,5,6]. Intensive research is still being conducted on a large scale to determine the effect of alloy additions on the microstructure and properties of these materials. Due to the wide range of possible compositions, HEA designs are still an evolving field, and researchers are trying to develop new alloys for projected applications [6,7,8].

Among HEAs, the CoCrFeNi system [9] warrants special attention due to its simple face-centered cubic (FCC) solid solution structure, good thermal stability, and favorable mechanical properties, all of which were combined with exceptional corrosion resistance. It is often the starting point for further modifications of the chemical composition aimed at improving specific performance parameters. Often, the CoCrFeNi alloy is modified by the addition of alloying elements such as Nb, Ti, W, or Zr [10,11,12]; however, recently, there has been a growing interest in the field of HEAs with vanadium (V), which is considered unique among alloying elements [13,14,15,16,17,18,19]. The uniqueness of V is manifested in its atomic volume, which is large in FCC-structured alloys and small in the case of the alloys with a body-centered cubic (BCC) structure. This leads to a significant volume mismatch in both crystal structures, resulting in strong strengthening effects in both FCC and BCC HEAs [14]. Significant strengthening combined with a loss of ductility in V-containing alloys has also been demonstrated as a result of the formation of the sigma (σ) phase [20], the amount of which is directly related to the mechanical properties and corrosion resistance of HEA. For this reason, careful optimization of the chemical composition is necessary because V acts as a strong σ-phase stabilizer, which may lead to undesirable effects in terms of the balance between the strength and ductility of the material [21,22]. The addition of V to the CoCrFeNiV_x_ (x = 0, 0.5, 1, 1.5) alloy increased the tensile strength, hardness, and ductility while maintaining the single-phase FCC solid solution structure of the alloy.

Furthermore, it contributed to strengthening the solid solution, grain refinement, and reduction in Cr segregation in the alloy [23]. In CoCrFeNiV_x_ (x = 0, 0.25, 0.5, 0.7, 0.8, 0.9, and 1.0) prepared by copper mold casting, the formation of the σ phase effectively improved the mechanical properties of the alloys. With increasing V content, the compressive yield strength increased from 164 MPa (x = 0) to 1493 MPa (x = 1.0) [24]. In turn, in AlCoCrFeNi alloys, with the increase in the V content from x = 0 to 2.0, the crystal structure of the alloy is transformed from the FCC phase to the FCC + σ phase and then to the BCC structure, which confirms that the V element stabilizes the BCC structure [25]. Similarly, in the AlCoCrFeNiV_x_ alloy, the strengthening factor was the solid solution strengthening of the BCC matrix. As the V content increased from 0 to 1.0, previously unattainable compressive strength and plastic deformation values of 3297.8 MPa and 26.8%, respectively (for the AlCoCrFeNiV_0.2_ alloy), were achieved. The influence of V on increasing the hardness of the alloy and transforming its ferromagnetic properties into paramagnetic properties has also been observed [26]. The presence of V also increased the hardness by 33% and significantly reduced the frictional wear by 73% of the Al_0.7_CoCrFeNiV_0.5_ alloy obtained by induction melting. At the same time, modification of the alloy by adding V resulted in homogenization of the structure [27].

Typically, V is used in refractory HEAs with a BCC structure along with elements such as Nb, Mo, Ta, Ti, and Zr, where an increase in the V content substantially improves both the yield strength and ductility at room temperature. Furthermore, it increases the tetragonal shear modulus, ensuring that V improves the mechanical stability of the BCC phase and also increases the elastic modulus [28,29]. Vanadium as an alloying additive can also be used in HEAs for large-scale electrochemical water splitting, where it enhances electrical conductivity and electrochemical surface area, thereby accelerating the activity of the electrocatalytic hydrogen evolution reaction [30]. In turn, studies on the effect of V content on the in situ oxidation, high-temperature mechanical strength, and tribological properties of Al_0.5_CrFeNiV_x_ alloys have confirmed the potential for applications that require exceptional mechanical strength, wear resistance, and thermal stability. In situ oxidation studies have demonstrated that an increased V content accelerates the oxidation process, providing direct evidence of vanadium oxide melting at 700 °C. As a result, liquid oxide phases are formed on the surface of the HEA, the presence of which during tribological contact at elevated temperature contributes to a reduction in friction and promotes the formation of thicker and more complex oxide layers on the wear surface, which in turn leads to an improvement in the wear resistance of HEA [31]. V also enhances the anticorrosion properties of high- and medium-entropy alloys (MEAs). When added to CrFeCoNi, it resulted in a noticeably broad passivation region of approximately 0.763 V from the corrosion potential, which was related to the formation of vanadium oxide during repassivation and the formation of a passive film with a multilayer structure [32].

The base alloy CoCrFeNi was also doped with metalloid elements, including silicon [33,34,35,36,37,38,39]. It has been shown that the addition of Si simultaneously increases the strength and ductility of CoCrFeNi HEA as a result of the precipitation of intermetallic compounds. According to the Hume-Rothery rule, the introduction of Si into alloys with the FCC structure leads to the formation of a substitutional solid solution [33]. Also, in [40], an increase in yield strength to 65%, tensile strength to 34%, and ductility to 15% of the CoCrFeNi alloy with Si addition was reported, attributed to the synergistic effect of the solid solution strengthening and reduced stacking fault energy. These HEAs also hold great promise for a variety of applications, including as an alternative to conventional nuclear radiation shielding materials [41]. Furthermore, it has been demonstrated that non-metallic elements such as Si can significantly improve the wear, oxidation, and corrosion resistance, as well as the soft magnetic properties of HEAs [42].

Despite numerous reports on CoCrFeNi alloys with Si or V additions, there are no studies available in the literature on CoCrFeNi systems containing both elements simultaneously. The CoCrFeNiSiV_x_ alloy proposed in this work represents a new, previously unexplored configuration that can combine the advantages of Si and V. Investigating such compositions will address a gap in the current literature on the design of HEAs. The synergistic effect of Si and V additions is expected to result in beneficial material properties, especially regarding future applications. In this work, CoCrFeNiSiV_x_ (x = 0.25; 0.5; 0.75; 1.0) high-entropy alloys, prepared in the form of ingots, were tested to determine their structure and selected mechanical properties, as well as their corrosion resistance.

## 2. Materials and Methods

The investigated HEAs were prepared in the form of ingots, with the use of high-purity elements (99.99 wt.%). As-cast alloys were obtained using the induction melting method, with the use of an NG-40 induction generator (Łukasiewicz Research Network, Gliwice, Poland), under a protective argon atmosphere. The alloys were solidified with a low cooling rate using Al_2_O_3_ crucibles.

An analysis of the alloys’ phase compositions was conducted with the use of an X-ray diffractometer, Rigaku MiniFlex 600 (Rigaku, Tokyo, Japan), equipped with a copper tube (Cu K_α_, λ = 0.15406 nm) and a D/TEX strip detector. Phase analysis and whole-powder-pattern fitting (WPPF) were conducted using dedicated PDXL2 software (ver. 2.9.2.0) and the PDF-2 database. The microstructure of the studied alloys was analyzed using a Zeiss Supra 35 scanning electron microscope (SEM) (Zeiss, Oberkochen, Germany) with the use of a back-scattered electron detector (BSD) for imaging. The chemical element maps and point analyses were performed using the EDX spectrometer (EDAX, Mahwah, NJ, USA).

To characterize the corrosion resistance of the alloys, electrochemical measurements were performed in a 3.5% sodium chloride solution at a temperature of 25 °C. The Autolab 302 N potentiostat (Metrohm-Autolab, Utrecht, The Netherlands), equipped with a three-electrode measuring system, was used for the study. The instrument was controlled with the NOVA 1.11 software. A saturated calomel electrode (SCE) (Hanna Instruments, Padua, Italy) was used as the reference electrode, a platinum wire as the counter electrode, and the material being tested as the working electrode. The changes in the open-circuit potential (*E*_OCP_) were recorded first. Electrochemical impedance spectroscopy measurements were subsequently performed at open-circuit potentials. The impedance data were collected over a frequency range of 10^−2^ to 10^5^ Hz, using a perturbation signal with an AC amplitude of 5 mV. Subsequently, potentiodynamic polarization measurements were conducted, in the range of –400 mV to 400 mV, with a scan rate of 1 mV/s. The corrosion potential (*E*_corr_), corrosion current density (*j*_corr_), and polarization resistance (*R*_p_) were subsequently determined using the Tafel extrapolation method.

Hardness measurements were performed with the use of Future Tech FM-7000 Vickers hardness instrument (Future Tech, Tokyo, Japan) with a load of 1000 g for 15 s. Tribological tests were conducted using the pin-on-disk method using a tribometer (CSM Instruments, Peseux, Switzerland). The radius of the wear track was 1.5 mm, and the counter-sample was a ball made of Al_2_O_3_ (d = 6 mm). The linear speed was 0.01 m/s, and a load of 10 N was applied. The surface morphology of the wear tracks was observed using a Zeiss Supra 35 SEM, with secondary electron (SE) detection employed for imaging.

## 3. Results and Discussion

### 3.1. XRD Analysis

**Figure 1:**
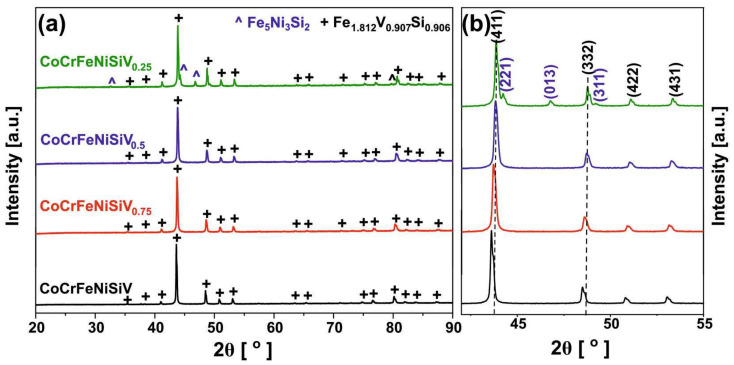
X-ray diffraction patterns of the CoCrFeNiSiV_x_ (x = 0.25; 0.5; 0.75; 1.0) HEAs (**a**) with an enlarged view highlighting the diffraction peak shifts associated with changes in the lattice parameters (**b**).

Figure 1 shows the X-ray diffraction patterns obtained for the CoCrFeNiSiV_x_ alloys in the as-cast state. The absence of the typical FCC phase was confirmed for all samples. The CoCrFeNiSiV_0.5_, CoCrFeNiSiV_0.75,_ and CoCrFeNiSiV alloys can be described as being single-phase, with the structure corresponding to the Fe_1.812_V_0.907_Si_0.906_ (PDF-01-075-2628) intermetallic phase characterized by a body-centered cubic structure (space group: $I\bar{4}3m$). The diffraction pattern obtained for the CoCrFeNiSiV_0.25_ alloy manifested the presence of the same phase, although simultaneously minor reflections corresponding to the Fe_5_Ni_3_Si_2_ (PDF-01-077-9327) intermetallic phase (cubic structure; space group: P2_1_3) were also observed. Currently, the difference in relative intensity clearly indicates that the intermetallic phase with a structure analogous to the Fe_1.812_V_0.907_Si_0.906_ phase represents a main constituent. The formation of a dual-phase structure consisting of two intermetallic phases was previously observed—cubic TM_3.921_Si_1.079_ (TM: Co, Cr, Fe, Ni) as the main constituent and the minor volume content of orthorhombic Ni_2_Si for the equimolar CoCrFeNiSi alloy [34]. The stabilization of the intermetallic phases can be attributed to the addition of silicon, which has a smaller atomic radius and a highly negative mixing enthalpy with the other constituent elements [43,44]. The shift in the diffraction peaks with increasing vanadium concentration was observed in the analyzed system (Figure 1b). This shift was related to changes in the lattice parameters, which were also confirmed by the WPPF analysis (Table 1). As can be seen, with increasing V content, the unit cell parameters (a, b, and c) increase from 8.7396(4) Å to 8.7870(4) Å, a value close to the theoretical value of 8.8120 Å.

### 3.2. Microscopic Observations

**Figure 2:**
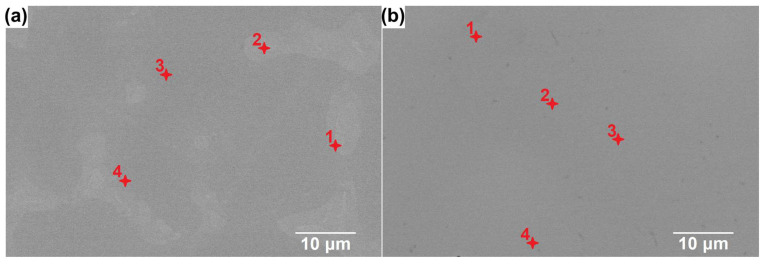
SEM-BSD images of the CoCrFeNiSiV_0.25_ (**a**) and CoCrFeNiSiV (**b**) alloys with the marked points of EDX analysis.

**Figure 3:**
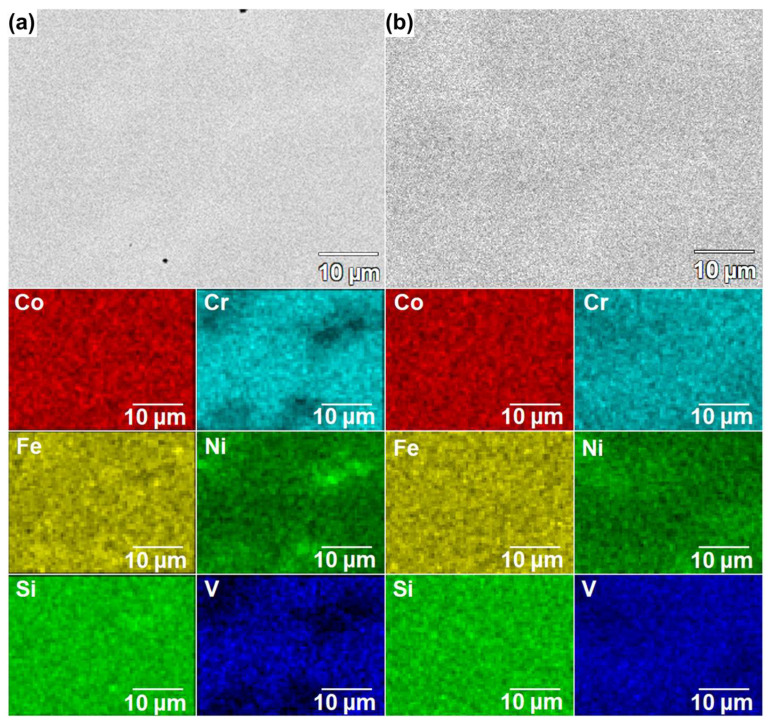
Microstructure image and results of EDX elemental distribution mapping for the CoCrFeNiSiV_0.25_ (**a**) and CoCrFeNiSiV_0.5_ (**b**) alloys.

**Figure 4:**
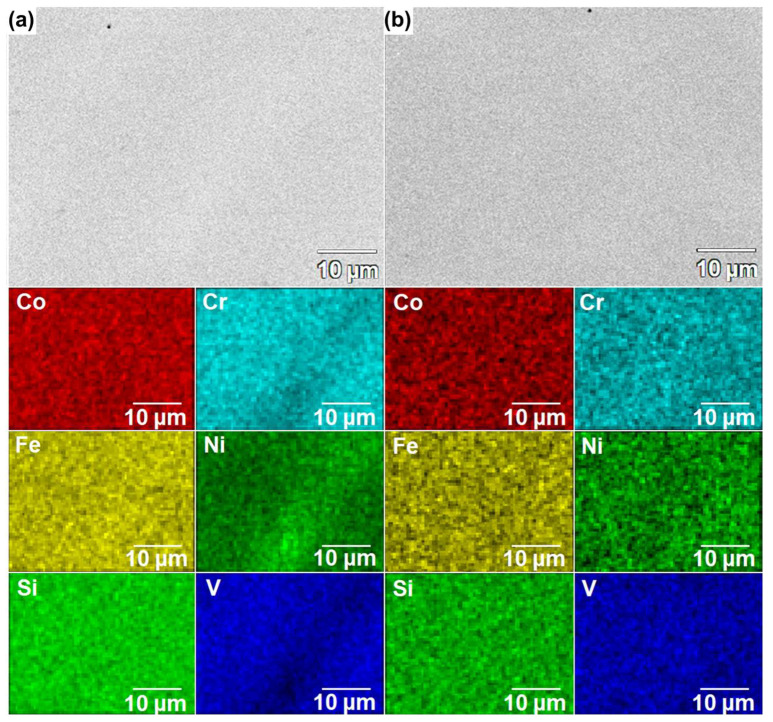
Microstructure image and results of EDX elemental distribution mapping for the CoCrFeNiSiV_0.75_ (**a**) and CoCrFeNiSiV (**b**) alloys.

The observations were conducted using the SEM in the BSD mode (Figure 2), in parallel with EDX point analyses, to support the phase identification. The results of performed EDX point analyses (Table 2) indicated that the precipitates—corresponding to the brighter areas in the SEM-BSE images—are characterized by the increased Ni content, while being depleted of Cr and V (Figure 2a—points 1 and 2), which confirms the proposed Fe_5_Ni_3_Si_2_ phase. In turn, in the case of the CoCrFeNiSiV alloy, a homogeneous single-phase structure can be observed in the obtained SEM-BSE images (Figure 2b). To complement the structural analysis, 2D EDX maps of elemental distribution were collected for the as-cast CoCrFeNiSiV_x_ (x = 0.25; 0.5; 0.75; 1.0) alloys and are presented in Figure 3 and Figure 4. In the case of the CoCrFeNiSiV, the homogenous distribution of the elements was revealed (Figure 4b), confirming the previous results. The occurrence of a pronounced elemental segregation can be noticed for CoCrFeNiSiV_0.25_, congruently with the results of XRD analysis, which indicated its dual-phase structure. Simultaneously, although the X-ray diffraction patterns recorded for the CoCrFeNiSiV_0.5_ and CoCrFeNiSiV_0.75_ alloys have shown only the presence of reflections deriving from the Fe_1.812_V_0.907_Si_0.906_-type phase (Figure 1a), the occurrence of an analogous elemental segregation as in the case of the alloy with lowest V content allows us to infer their dual-phase structure, with the second phase present in a low volume fraction, being below the detection limit of the XRD method. A heterogeneous distribution of Cr, Ni, and V allows the distinction of two different regions in the alloy’s microstructure. The first, corresponding to the brighter areas in the SEM images, is depleted of Cr and V, simultaneously being enriched with Ni. Consequently, these areas can be assigned to the off-stoichiometric Fe_5_Ni_3_Si_2_-type phase identified in the XRD patterns. The areas of darker shade were enriched with Cr and V and also characterized by a lower concentration of Ni. Accordingly, it can be related to the majority phase corresponding to the Fe_1.812_V_0.907_Si_0.906_-type phase with an off-stoichiometric composition. Concurrently, nearly uniform distributions of Co, Fe, and Si were observed. The occurrence of Ni and Cr segregation also characterized the CoCrFeNiSi alloy [34,36]. In the work of Guan et al. [36], the presence of a minor volume content (5 wt.%) of the intermetallic Ni(Fe,Co)_2_Si phase was noticed at the grain boundaries of the dominant Fe_2.5_Ni_1.5_Si-type phase. In turn, the appearance of regions enriched with Ni and Si, while Cr- and Fe-lean, was found for the CoCrFeNiSi described in the previous study [34].

In the case of the high-entropy transition metal alloy systems, the introduction of V usually stabilized the intermetallic σ phase [17,19,20,22,24,25]. In [22], describing the Al_0.1_CoCrFeNiV_x_ (where x = 0, 0.2, 0.4, 0.6, 0.8, 1.0) alloys, prepared with the use of the arc-melting method, the formation of a dual-phase structure comprising FCC solid solution and intermetallic σ phase was observed for V concentrations exceeding 4.65 at.%. In the case of the equimolar AlCoCrFeNiV alloy, the microstructure was described as consisting of intertwined stripes of both phases. The σ phase was found to be enriched with Cr and V, but depleted with Ni. Similarly, in the case of the CoCrFeNi alloy, the addition of V contributed to the precipitation of the intermetallic (Cr,V)-rich σ phase [20]. Salischev et al. [20] reported that the microstructure of the CoCrFeNiV alloy consists of the major volume fraction of the eutectoid-like structures with the alternating σ phase and FCC solid solution plates, alongside FCC solid solution regions at the grain boundaries.

### 3.3. Differential Thermal Analysis

**Figure 5:**
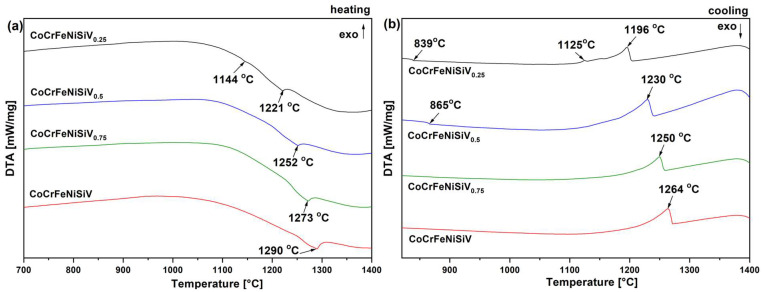
DTA curves of the CoCrFeNiSiV_x_ (x = 0.25; 0.5; 0.75; 1.0) alloys after heating (**a**) and cooling (**b**) at 20 °C/min.

The DTA heating and cooling curves of the CoCrFeNiSiV_x_ alloys are presented in Figure 5. For the CoCrFeNiSiV_0.25_ alloy, the appearance of two thermal events was observed on the heating curve—a thermal event with a low-heat-flow signal at 1144 °C, followed by a more pronounced peak at 1221 °C. In the case of cooling, the observed thermal events correspond to changes during heating. An event with a high-heat-flow signal was observed at 1196 °C, followed by a minor exothermic peak at 1125 °C. Furthermore, a low-heat-flow event was observed at a temperature of 839 °C. This thermal peak is likely related to the Fe_5_Ni_3_Si_2_-type phase observed in the structure of the CoCrFeNiSiV_0.25_ alloy. According to Maji et al. [45], in the Fe-Mn-Si-Cr-Ni stainless steel, the field of occurrence of the austenite and the Fe_5_Ni_3_Si_2_ phase in the temperature range of 700–1000 °C was reported. For the CoCrFeNiSiV_0.5_ alloy, a low thermal event was also observed at 865 °C. The higher transformation temperature, compared to the CoCrFeNiSiV_0.25_ alloy, may be related to a higher V content that has a high melting point (1890 °C) [46]. Vanadium was more uniformly distributed in the CoCrFeNiSiV_0.5_ alloy structure compared to the CoCrFeNiSiV_0.25_ alloy. For the other alloys, no thermal events were identified in the range of 700–1000 °C, which is due to a low share of the Fe_5_Ni_3_Si_2_-type phase in the CoCrFeNiSiV_0.75_ alloy and its absence in the equimolar CoCrFeNiSiV alloy. In case of CoCrFeNiSiV_0.5_, CoCrFeNiSiV_0.75_, and CoCrFeNiSiV alloys, pronounced thermal events were directed towards higher temperatures with increasing V content, corresponding to the temperatures of 1252, 1273, and 1290 °C, respectively, for the CoCrFeNiSiV_0.5_, CoCrFeNiSiV_0.75_, and CoCrFeNiSiV alloys. Similarly, the presence of only one exothermic peak can be observed in the case of the corresponding cooling curves.

### 3.4. Electrochemical Investigations

**Figure 6:**
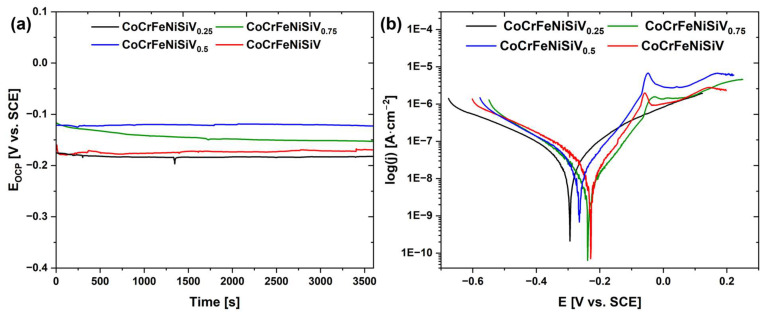
Time dependence of the open-circuit potential (**a**) and potentiodynamic polarization curves (**b**) for the as-cast CoCrFeNiSiV_x_ (x = 0.25; 0.5; 0.75; 1.0) alloys recorded in the 3.5% NaCl solution at 25 °C.

To describe the influence of V addition on the corrosion resistance of the CoCrFeNiSiV_x_ alloys, electrochemical tests were carried out with the use of the potentiodynamic polarization method in the 3.5% NaCl solution, at 25 °C. The recorded time dependence of the open-circuit potentials is presented in Figure 6a, while Figure 6b shows the recorded potentiodynamic polarization curves. The quantitative parameters of the open-circuit potential, corrosion potential, polarization resistance, and corrosion current density are summarized in Table 3. The highest open-circuit potential value was achieved by the CoCrFeNiSiV_0.5_ alloy (−0.123 V). The presence of the passive regions in the polarization curves recorded for CoCrFeNiSiV_0.5_, CoCrFeNiSiV_0.75_, and CoCrFeNiSiV alloys was observed, with the passive current density values decreasing with the V content (Figure 6b). The beneficial effect of V on the passivation ability of the HEAs, related to the formation of vanadium oxide (V_2_O_5_), was confirmed in earlier works [18,32,47]. The corrosion current density values decreased with increasing V content, indicating an improvement in the alloy corrosion resistance, further evidenced by the positive shift in the corrosion potential values. Concurrently, a substantial increase in the polarization resistance was observed, with the V content being a notable factor, yielding the highest value in the case of the equimolar CoCrFeNiSiV alloy (514.3 kΩcm^2^).

Compared with the CoCrFeNiSi alloy characterized in the previous work [34], a positive influence of V addition on the polarization resistance can be noticed. At the same time, all the studied alloys except CoCrFeNiSiV_0.25_ also exhibited substantially lower *j*_corr_ values in comparison to the equimolar CoCrFeNiSi alloy (1.77 μA/cm^2^), indicating a decreased corrosion rate. Earlier works [18,47] also investigated the influence of V on the corrosion resistance of the HEAs, although for different alloying systems and manufacturing methods. Souto et al. [18] studied the corrosion resistance of the AlCoCrFeNiV and AlCo_0.5_CrFeNiV_0.5_ alloys in the 3.5% NaCl solution, revealing the beneficial effect of the higher V content on the corrosion resistance. The AlCoCrFeNiV alloy was characterized by a one order of magnitude lower corrosion current density value (0.93 μA/cm^2^) and a substantially more noble corrosion potential (−0.052 V), which was attributed to enhanced passive film protective abilities and a more homogeneous microstructure.

In turn, in [47], the positive influence of moderate V addition on the corrosion resistance of the Al_0.5_Cr_0.9_FeNi_2.5_V_x_ (x = 0, 0.2, 0.4, 0.6, 0.8, 1.0) alloys was observed. Simultaneously, the introduction of V in higher concentrations contributed to the occurrence of galvanic corrosion, which is related to the formation of the (Cr,V)-rich phase. Compared to the CoCrFeNiSiV_x_ alloys described in this work, less favorable values of the corrosion current density were obtained, ranging from 2.276 μA/cm^2^ for the Al_0.5_Cr_0.9_FeNi_2.5_V_1.0_ alloy to 3.828 μA/cm^2^ in the case of the Al_0.5_Cr_0.9_FeNi_2.5_V_0.8_ alloy.

**Figure 7:**
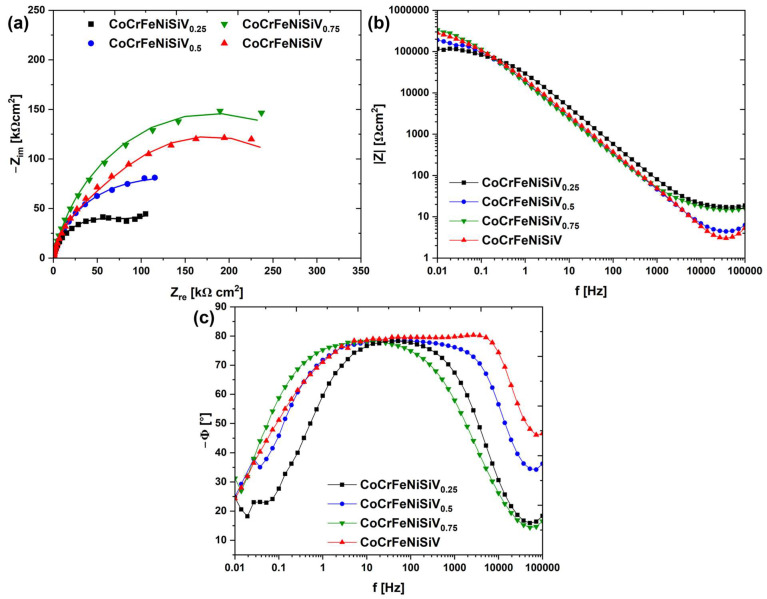
Experimental EIS spectra: Nyquist plots with solid lines representing the fitting results (**a**), Bode phase angle plots (**b**), and Bode modulus plots (**c**) for the CoCrFeNiSiV_x_ alloys (x = 0.25; 0.5; 0.75; 1.0) recorded in 3.5% NaCl solution at 25 °C.

To investigate the passivation behavior of the CoCrFeNiSiV_x_ alloys, electrochemical impedance spectroscopy (EIS) measurements were conducted. Figure 7 presents the Nyquist and Bode plots recorded at the open-circuit potential. The Nyquist plots are characterized by the presence of unfinished semicircles, whose diameter can be associated with the resistance to charge transfer at the electrode interface [48]. The diameter of the semicircle gradually increased with V content, reaching a maximum for the CoCrFeNiSiV_0.75_ alloy (Figure 7a), and subsequently decreased slightly for the CoCrFeNiSiV alloy. For the CoCrFeNiSiV_0.5,_ CoCrFeNiSiV_0.75_, and CoCrFeNiSiV alloys, phase angle values of nearly −80° were maintained in the broad range of frequencies (Figure 7c), which suggests the considerable stability of the passive film formed on their surface [49].

**Figure 8:**
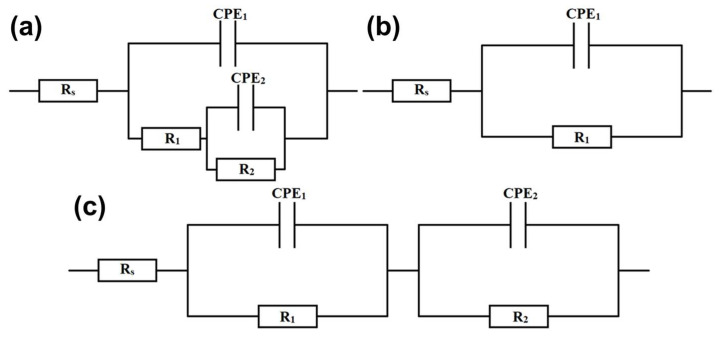
Proposed equivalent electric circuit for the CoCrFeNiSiV_0.25_ (**a**), CoCrFeNiSiV_025_ and CoCrFeNiSiV_0.75_ (**b**), and CoCrFeNiSiV (**c**) alloys in 3.5% NaCl solution at 25 °C.

The obtained impedance spectra were subsequently analyzed, using the electric equivalent circuits (EECs) shown in Figure 8. The EEC used in the case of CoCrFeNiSiV_0.25_ consists of the solution resistance (*R*_s_), passive film resistance (*R*_1_), charge transfer resistance (*R*_2_), constant phase elements representing the passive film (*CPE*_1_), and double-layer capacitance (*CPE*_2_) [50]. The passive film is considered to have a non-homogenous structure, related to the occurrence of elemental segregation. The presence of a significant volume content of the Fe_5_Ni_3_Si_2_-type phase, depleted of Cr and V, leads to the formation of a less stable passive film in precipitation areas, increasing the susceptibility to localized corrosion. A constant phase element was used to account for the frequency dispersion of the electrode capacitance, which can be related to the surface inhomogeneity [51]. The impedance of the constant phase element was calculated using Equation (1):

(1) $$Z\left(\omega \right)={Z_{0}\left(i\omega \right)}^{-n}$$

where *Z*_0_ represents a constant connected with the electrode capacitance, *i* is the imaginary unit, *ω* represents the angular frequency [rad/s], and *n* is the constant phase exponent [52]. Depending on the *n* value, the constant phase element represents the pure capacitance (*n* = 1), Warburg impedance (*n* = 0.5), or pure resistance (*n* = 0) [53].

The proposed EEC was used to fit the spectra obtained for the CoCrFeNiSiV_0.5_ and CoCrFeNiSiV_0.75_ alloys (Figure 8b), assuming the formation of a homogeneous passive film, resulting from the more homogenous elemental distribution. The circuit involved solution resistance (*R*_s_), passive film resistance (*R*_1_), and a constant phase element, representing the passive film capacitance (*CPE*_1_) [54]. For the equimolar CoCrFeNiSiV alloy, considering the presence of dual broadening on the Bode phase angle plot (Figure 7c), the EEC that encompasses two time constants was utilized (Figure 8c). In this case, *R*_1_ and *CPE*_1_ corresponded to the resistance and capacitance of the outer layer of the passive film, while *R*_2_ and *CPE*_2_ represent the inner layer [55]. The formation of the passive film of the bilayer structure, with the outer layer enriched with vanadium and a nickel oxide, and an inner layer consisting of cobalt, chromium, and iron oxides, was previously observed in the case of the Co_29.82_Cr_9.90_Fe_45.48_Ni_4.92_V_9.88_ medium-entropy alloy [32]. The cation-selective characteristics of the vanadium-rich oxide layer can contribute to enhanced resistance to pitting corrosion, inhibiting inward diffusion of the chloride ions [32].

The obtained electrochemical parameters after impedance spectra analysis are summarized in Table 4. The polarization resistance can be derived from the fitting results as *R*_p_ = *R*_1_ + *R*_2_ [10,50]. The substantially higher polarization resistance values obtained for the CoCrFeNiSiV_0.75_ and CoCrFeNiSiV alloys confirmed the beneficial effect of increased V content on the passive film protective abilities. However, although the CoCrFeNiSiV_0.75_ alloy exhibits the highest impedance values, suggesting the formation of the passive film with the most favorable protective abilities, higher corrosion current density and lower corrosion potential obtained in the potentiodynamic polarization measurements indicate its greater susceptibility to corrosion initiation. This discrepancy may be related to microstructural effects such as elemental segregation resulting in local electrochemical potential differences, and discontinuities or porosity of the passive layer. In particular, the heterogeneity of Cr and V distribution, with their lower concentration in the Fe_5_Ni_3_Si_2_-type phase areas, may contribute to the formation of a less stable passive film and higher vulnerability to localized corrosion. Consequently, despite its apparently better impedance parameters, this sample may be less corrosion-resistant than the CoCrFeNiSiV alloy, characterized by a single-phase structure and homogenous elemental distribution [56,57,58]. However, the values of the constant phase exponent obtained for the CoCrFeNiSiV_0.5_, CoCrFeNiSiV_0.75_, and CoCrFeNiSiV alloys still suggest the formation of a relatively homogenous passive film [48]. In turn, the significant value of the double-layer capacitance characterizing the CoCrFeNiSiV_0.25_ indicates an increased surface charge density, which enhances the movement of ions across the double layer and intensifies the corrosion process [50].

### 3.5. Hardness

**Figure 9:**
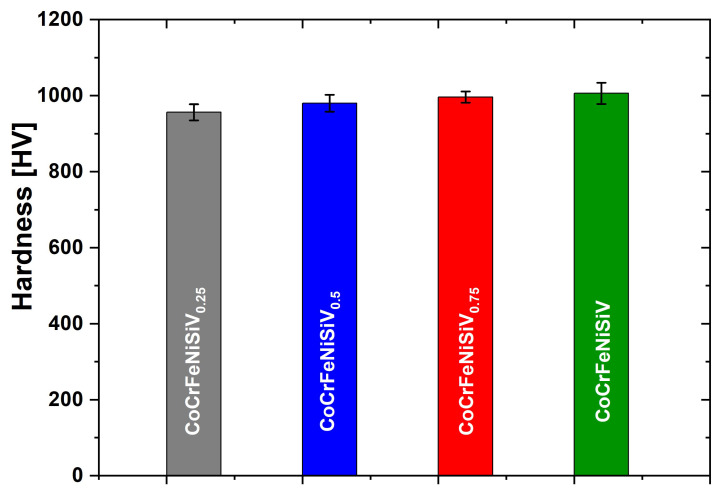
Variation in Vickers hardness of CoCrFeNiSiV_x_ (x = 0.25; 0.5; 0.75; 1.0) alloys.

The results of the hardness measurements of the CoCrFeNiSiV_x_ (x = 0.25; 0.5; 0.75; 1.0) alloys, along with the standard deviations, are presented in Figure 9. A visible correlation exists between the V content and the hardness of the studied alloys. With the increasing V content, the hardness of the alloys increased from 956 HV (0.25 V), 980 HV (0.5 V), and 996 HV (0.75 V) to 1006 HV for the equimolar CoCrFeNiSiV alloy, respectively. Therefore, it can be concluded that the addition of V significantly increases the hardness of the alloys in relation to the base FeCoCrNiSi alloy prepared by vacuum arc melting, for which the hardness of 653.71 HV was obtained [42]. The increase in hardness of the CoCrFeNiSiV_x_ alloy (x = 0.25; 0.5; 0.75; 1.0) can be attributed to the presence of intermetallic phases with varying contributions. In the case of the CoCrFeNiSiV_0.25_ alloy, characterized by the lowest hardness value, the highest content of the Fe_5_Ni_3_Si_2_-type phase is noticeable, alongside the main structure constituent, which was the Fe_1.812_V_0.907_Si_0.906_-type phase. In turn, the CoCrFeNiSiV HEA, which exhibited the highest hardness value, had a relatively uniform microstructure, and only the reflections deriving from the Fe_1.812_V_0.907_Si_0.906_-type phase were observed on the XRD pattern. Concurrently, the relatively weak effect of increasing V content on hardness may be related to the rather very low volume share of the Ni-enriched second phase—even in the case of the CoCrFeNiSiV_0.25_ alloy. A similar relationship between the improvement in mechanical properties and the volume fraction of the phases was revealed in [39]. The correlation of mechanical properties with the phase composition of the CoCrFeNiV system was also observed in [20]. In this case, the increase in microhardness is attributed to the formation of the σ phase caused by the addition of V to the CoCrFeNi alloy. In turn, in the study [23] on the effect of V on CoCrFeNiV_x_ (x = 0, 0.5, 1, 1.5) HEA, an improvement in the hardness of the alloy was found without changing its phase structure.

In the work of Dong et al. [26], the improvement in the hardness with increasing V content in the AlCoCrFeNiV_x_ alloy (x = 0, 0.2, 0.5, 0.8, 1.0) was primarily due to the increase in the lattice distortion and related solid solution strengthening, as indicated by the change in the position of (110) diffraction peaks to lower 2θ values. A different effect of V content on hardness results was shown by Chen et al. [25], where a high-entropy alloy of Al_0.5_CoCrCuFeNiV_x_ (x = 0 to 2.0) was studied. At a V content below 0.4, a slight effect of V on the hardness was observed. With increasing V content, a sharp increase in the hardness values was recorded with a maximum (640 HV) at x ≤ 1.0. An increase in the V content from 1.0 to 1.2 resulted in a decrease in hardness to 576 HV. With a further increase in the V content, the hardness remained almost unchanged, which was explained by the evolution of the microstructure. In this work, the simultaneous introduction of Si and V additions to the CoCrFeNi alloy enabled the achievement of favorable mechanical properties of the material. A significant increase in the hardness of the equimolar CoCrFeNiSiV alloy was observed, compared to the base CoCrFeNi alloy (160 HV) [20,39].

### 3.6. Wear Resistance

**Figure 10:**
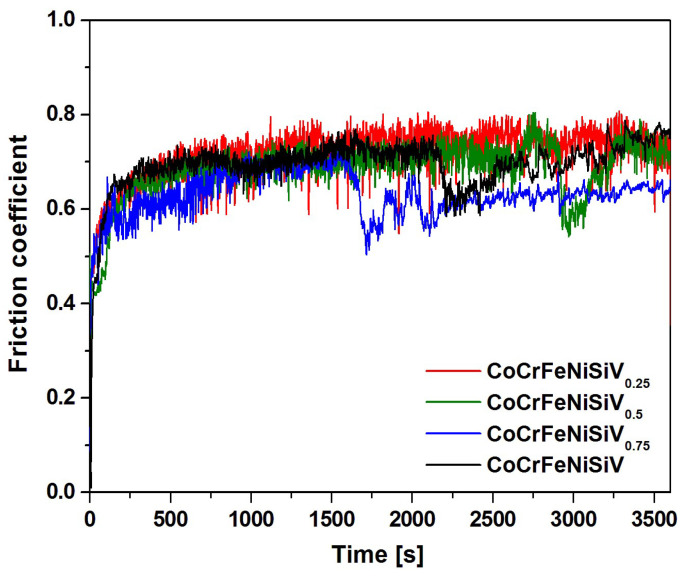
Changes in the friction coefficient as a function of time for the CoCrFeNiSiV_x_ (x = 0.25; 0.5; 0.75; 1.0) alloys.

**Figure 11:**
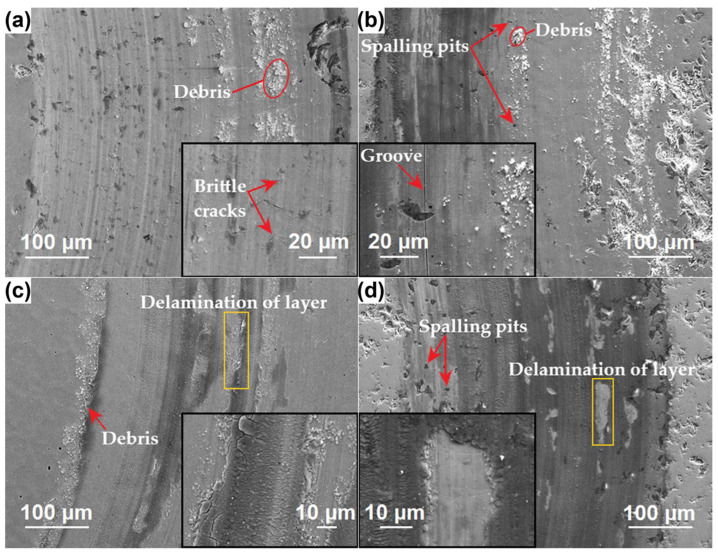
Surface morphologies of wear tracks of the CoCrFeNiSiV_0.25_ (**a**), CoCrFeNiSiV_0.5_ (**b**), CoCrFeNiSiV0_.75_ (**c**), and CoCrFeNiSiV (**d**) alloys.

To describe the wear resistance of the CoCrFeNiSiV_x_ alloys, tribological tests using the pin-on-disk method were performed. Figure 10 shows the recorded variation in the friction coefficient as a function of time. The SEM images of the worn surface morphology after pin-on-disk tests are presented in Figure 11. In the case of the CoCrFeNiSiV_0.25_ alloy, the course of the curve was characterized by a rapid increase in the initial 500 s of the test, followed by its subsequent stabilization. In contrast, for alloys with a higher V content, the fluctuations occurred after the period of stable wear. A sharp decrease in the friction coefficient was observed, which can be related to the detachment of the debris accumulated on the alloy surface. Subsequently, a gradual increase in the friction coefficient was observed. The appearance of large fluctuations can be connected with the periodic accumulation and detachment of the wear products [59]. Noticeably, for the CoCrFeNiSiV_0.75_ alloy, the friction coefficient remained at lower values after the observed sharp decrease, which may result from the enhanced oxide layer formation (Figure 11c). The lowest average friction coefficient was observed for the CoCrFeNiSiV_0.75_ alloy (0.63 ± 0.06), whereas CoCrFeNiSiV_0.5_ and CoCrFeNiSiV exhibit similar values of 0.68 ± 0.07 and 0.70 ± 0.06, respectively. The least favorable value of the friction coefficient was observed in the case of the CoCrFeNiSiV_0.25_ alloy (0.72 ± 0.07).

Lin et al. [43] investigated the wear resistance of CoCrFeMnNiSi_x_ coatings (where x = 0, 0.4, 0.8, 1.2, and 1.6) prepared using the laser cladding method. Ball-on-plate tests were conducted with a load of 30 N and the Si_3_N_4_ counter-sample at 25 °C. The positive effect of the increase in silicon content on the reduction in the friction coefficient was revealed, which was related to the transformation of the FCC solid solution into the BCC solid solution with higher hardness, and the formation of silicide intermetallic phases. CoCrFeMnNiSi_0.4_, CoCrFeMnNiSi_0.8_, and CoCrFeMnNiSi_1.2_ coatings exhibited a friction coefficient in the range of 0.71-0.73, similar to the results obtained in this work for the CoCrFeNiSiV_0.25_, CoCrFeNiSiV_0.5_, and CoCrFeNiSiV alloys. Concurrently, the lowest value obtained in the work [43] for CoCrFeMnNiSi_1.6_ (±0.65) was slightly less favorable than the value exhibited by the CoCrFeNiSiV_0.75_ alloy described in this study.

The worn surface of the CoCrFeNiSiV_0.25_ alloy (Figure 11a) is characterized by numerous microcracks perpendicular to the sliding direction, as well as cavities, which may result from brittle spalling. Additionally, the grooves, indicative of abrasion wear and deposits of loose, oxidized debris, were observed. Therefore, the wear of abrasion and fatigue as the main wear mechanisms was described. The increase in the V content noticeably influenced the wear mechanisms. The appearance of dark areas on the surface of the worn tracks was observed, which can be associated with increased surface oxidation [44]. The abrasive wear was alleviated with an increasing content of V, which can be attributed mainly to the extended surface coverage by the oxide layer and, to a lesser extent, the slight increase in the hardness. Both V and Si are conducive to the formation of oxide layers, which can be further enhanced by the generation of friction heat, especially in dry friction conditions [27,43,44,60]. The generation of dense surface layers can contribute to an improvement in tribological properties, decreasing the friction coefficient and reducing direct contact between the sliding surfaces [43]. Concurrently, the large brittleness of these oxide layers made them prone to cracking and delamination (Figure 11d), resulting in the formation of loose debris that accumulated on the edges of the wear track (Figure 11c). The debris particles may also have contributed to the occurrence of three-body wear—evidenced by signs of micro-cutting wear in the case of CoCrFeNiSiV_0.5_ (Figure 11b). The spalling pits almost disappear in the case of the CoCrFeNiSiV_0.75_ alloy (Figure 11c), which may also be a reason behind its reduced friction coefficient. In contrast, CoCrFeNiSiV_0.5_ and CoCrFeNiSiV were characterized with pronounced spalling pits.

In the case of the CrFeNiTiSi alloy described in [44], the addition of silicon contributed to the generation of the oxide layer during friction, which beneficially influenced the resistance to wear. Similarly, the positive effect of increased silicon content on oxide layer formation was one of the factors behind the recorded low friction coefficient and diminished wear ratio recorded in the case of CoCrFeMnNiSi_x_ alloy coatings [43]. What is more, Zhang et al. [60] characterized the tribological properties of the equimolar CoCrFeNiV alloy obtained through mechanical alloying and vacuum hot pressing. The high V content contributed to the formation of the intermetallic σ phase, which beneficially affected the hardness and wear resistance. At room temperature, the coexistence of abrasive, mild adhesive wear, delamination, and oxidative wear was identified. The beneficial influence of V addition on the Al_0.7_CoCrFeNi alloy wear resistance was revealed in [27]. The changes in the phase structure and the related increase in the hardness, resulting from the introduction of V, alleviated the occurrence of adhesive wear and contributed to the higher resistance to abrasion.

## 4. Conclusions

The CoCrFeNiSiV_0.25_ alloy was characterized by a dual-phase structure consisting of the Fe_1.812_V_0.907_Si_0.906_ intermetallic phase with the presence of a minority Fe_5_Ni_3_Si_2_-type phase. The increase in the vanadium content enabled the obtaining of a homogeneous intermetallic phase structure in the case of the equimolar CoCrFeNiSiV alloy. The lattice parameters of this phase change with the increasing vanadium content from 8.7396(4) Å (for the CoCrFeNiSiV_0.25_ alloy) to 8.7870(4) Å (for the CoCrFeNiSiV alloy). The results of the potentiodynamic polarization measurements revealed the beneficial effect of V on the corrosion resistance of the investigated alloys. The equimolar CoCrFeNiSiV alloy is characterized by the highest corrosion resistance, which can be attributed to its most homogeneous elemental distribution. The EIS study indicated a positive influence of the higher content of V on the passive film’s protective abilities. The hardness of the CoCrFeNiSiV_x_ alloys increased with V content, achieving the highest value of 1006 HV for the equimolar alloy. Simultaneously, moderate V addition (x = 0.75) allowed for obtaining the most favorable friction coefficient of 0.63 ± 0.06. The abrasive, fatigue, and oxidative wear mechanisms were identified as the main wear mechanisms of the CoCrFeNiSiV_x_ alloys.

## Figures and Tables

**Table 1:** Whole-powder-pattern fitting (WPPF) results for CoCrFeNiSiV_x_ (x = 0.25; 0.5; 0.75; 1.0) alloys (*R*_wp_—weighted profile R-factor, *R*_pr_—profile R-factor, *R*_exp_—expected R-factor, *χ*^2^—goodness-of-fit indicator).

Alloy	Identified Phases	Space Group	Lattice Parameters	Statistical Quality Indices			
				*R*_wp_ (%)	*R*_pr_ (%)	*R*_exp_ (%)	*χ* ^2^
CoCrFeNiSiV_0.25_	Fe_1.812_V_0.907_Si_0.906_	$I\bar{4}3m$	a = b = c = 8.7396(4) Å; α = β = γ = 90°	1.31	1.00	1.47	0.80
	Fe_5_Ni_3_Si_2_	$P2_{1}3$	a = b = c = 6.1289(4) Å; α = β = γ = 90°				
CoCrFeNiSiV_0.5_	Fe_1.812_V_0.907_Si_0.906_	$I\bar{4}3m$	a = b = c = 8.7622(3) Å; α = β = γ = 90°	1.35	1.06	1.55	0.87
CoCrFeNiSiV_0.75_	Fe_1.812_V_0.907_Si_0.906_	$I\bar{4}3m$	a = b = c = 8.7794(10) Å; α = β = γ = 90°	1.35	1.04	1.53	0.77
CoCrFeNiSiV	Fe_1.812_V_0.907_Si_0.906_	$I\bar{4}3m$	a = b = c = 8.7870(4) Å; α = β = γ = 90°	1.41	1.07	1.56	0.81

**Table 2:** Chemical composition of different regions in the microstructure of the as-cast CoCrFeNiSiV_0.25_ and CoCrFeNiSiV alloys based on the results of EDX point analysis.

Alloy	Point	Content [at.%]					
		Co	Cr	Fe	Ni	Si	V
CoCrFeNiSiV_0.25_	1	15.6	9.6	12.4	36.3	23.9	2.2
	2	17.7	14.9	20.0	24.7	19.6	3.1
	3	19.2	21.6	17.9	16.4	18.8	6.1
	4	18.6	21.0	18.3	17.1	19.1	5.9
CoCrFeNiSiV	1	16.7	17.9	16.4	13.8	16.0	19.2
	2	16.4	17.6	15.6	15.1	16.4	18.9
	3	16.8	17.4	16.4	14.2	16.4	18.8
	4	16.8	17.5	16.0	15.8	16.0	17.9

**Table 3:** Electrochemical parameters of the CoCrFeNiSiV_x_ (x = 0.25; 0.5; 0.75; 1.0) alloys in the 3.5% NaCl solution at 25 °C.

Alloy	*E*_OCP_ [V] (±0.01)	*E*_corr_ [V] (±0.01)	*R*_p_ [kΩcm^2^] (±0.1)	*j*_corr_ [μA/cm^2^] (±0.01)
CoCrFeNiSiV_0.25_	−0.182	−0.294	173.5	2.01
CoCrFeNiSiV_0.5_	−0.123	−0.264	367.6	0.37
CoCrFeNiSiV_0.75_	−0.153	−0.237	465.2	0.32
CoCrFeNiSiV	−0.169	−0.228	514.3	0.17

**Table 4:** The fitted electrochemical parameters for the impedance data of the CoCrFeNiSiV_x_ (x = 0.25; 0.5; 0.75; 1.0) alloys in 3.5% NaCl solution at 25 °C.

Alloy	*R*_s_ [Ωcm^2^]	*R*_1_ [kΩcm^2^]	*CPE*_1_ [μΩ^−1^ cm^−2^ s^n^]	*n*_1_	*R*_2_ [kΩcm^2^]	*CPE*_2_ [μΩ^−1^ cm^−2^ s^n^]	*n*_2_
CoCrFeNiSiV_0.25_	16.2	81.5	5.9	0.88	152.0	46.8	0.61
CoCrFeNiSiV_0.5_	3.2	177.9	10.8	0.87	-	-	-
CoCrFeNiSiV_0.75_	14.6	367.5	11.9	0.86	-	-	-
CoCrFeNiSiV	1.3	51.4	15.4	0.89	274.8	20.9	0.89

## Data Availability

The data and materials generated and/or analyzed during the current study are available from the corresponding author upon reasonable request.
